# Comprehensive toxicity screening of Pazarsuyu stream water containing heavy metals and protective role of lycopene

**DOI:** 10.1038/s41598-022-21081-y

**Published:** 2022-10-05

**Authors:** Mahmut Doğan, Kültiğin Çavuşoğlu, Emine Yalçin, Ali Acar

**Affiliations:** 1 Department of Biology, Institute of Science, Giresun University, Giresun, Turkey; 2 Department of Biology, Faculty of Science and Art, Giresun University, Giresun, Turkey; 3 Department of Medical Services and Techniques, Vocational School of Health Services, Giresun University, Giresun, Turkey

**Keywords:** Environmental sciences, Environmental impact, Cytogenetics

## Abstract

In this study, heavy metal pollution in the Pazarsuyu stream of Giresun province and the protective role of lycopene against the toxicity caused by this pollution were investigated using the *Allium* test. Germination percentage, root length and weight gain as physiological markers of toxicity; mitotic index (MI), micronucleus (MN) and chromosomal aberrations (CAs) as genetic markers of toxicity; malondialdehyde (MDA) level, superoxide dismutase (SOD) and catalase (CAT) activities as biochemical markers of toxicity, and meristematic cell damages were used as anatomical markers. For this aim *Allium cepa* L. bulbs were divided into six groups and germinated for 72 h with 215 mg/L and 430 mg/L doses of lycopene, tap water and stream water. Heavy metals pollution was analyzed with ICP-MS and Fe > Sr > Ba > Be > Mo > Li were determined according to the rate of presence in the water samples of Pazarsuyu. As a result, germination-related parameters and meristematic cell proliferation of bulbs germinated with Pazarsuyu water samples decreased significantly. Germination percentage, root length and weight gain of the group treated with Pazarsuyu water samples were decreased 50%, 73% and 68%, respectively compared to control. In addition, MN and CAs frequencies, indicating the genotoxic effects, were increased and significant abnormalities were detected in MDA, SOD and CAT levels, which indicate the deterioration of antioxidant/oxidant balance. CA observed with high frequency was also confirmed by DNA fragmentation determined by the Comet test. Stream water application promoted anatomical damages such as epidermis and cortex cell damage, accumulation of some substances in cortex cells, flattened cell nucleus and non-apparent appearance of conduction tissue in root tip meristem cells. All these abnormalities observed in *A. cepa* root tip cells were associated with the presence of heavy metals in the water samples. Simultaneous application of lycopene with stream water reduced the effects of heavy metals and resulted in a dose-dependent improvement in all parameters studied. Lycopene application showed a protective role by providing an increase in germination parameters and MI, decrease in MN and CAs frequencies, and improvements in MDA, SOD and CAT activities. As a result, heavy metals detected in the water samples of Pazarsuyu stream caused multiple toxicities in the bio-indicator plant, and lycopene reduced this toxicity and recorded a protective role.

## Introduction

Climate change and rapid industrialization cause reduction and pollution in water resources. Water is one of the most important renewable resources for sustainable agricultural production, economic development and general well-being. It is also one of the best manageable natural resources due to its ability to divert, transport, store and recycle. Surface and underground water resources are vital in areas such as agriculture, hydropower, animal husbandry, industrial activities, forestry, fisheries, maritime and recreation. Despite this vital importance, the world's freshwater ecosystems constitute only 0.5% of the earth's surface, and within this percentile, the river constitutes 0.01% and is of great importance^1^. While the protection of freshwater resources is so essential for humanity, the pollution of natural water bodies by pathogenic microorganisms, radioactive substances, industrial and domestic wastes, organic and inorganic substances as a result of various activities negatively affects water quality. Common consequences of water pollution on ecosystems include the death of species, loss of biodiversity and loss of ecosystem services. The biggest cause of water pollution is chemicals discharged into natural water bodies as a result of human activities. The most common examples of this type of pollution are heavy metal ions such as Hg from mining activities, some nitrogen compounds used in agriculture, chlorinated organic molecules from sewage or water treatment plants, and acids formed as a result of various production activities^2^.

Today, with the increasing population, industrialization and technological developments, various pollutants reach aquatic environments and environmental pollution is increasing day by day. Heavy metals have an important place among these pollutants and constitute one of the most dangerous types of pollutants. Heavy metals with long biological half-life, persistence, non-biodegradability and potential for bioaccumulation in living organism tissues can cause serious health problems and economic losses. Heavy metals can damage the central nervous system, cardiovascular and gastrointestinal systems. They can also cause damage and loss of function in the lungs, kidneys, liver, endocrine glands, and bones^3–6^. In the literature, the toxic effects of the consumption of polluted water resources on human health have been reported. Mohod and Dhote^7^ determined that kidney, digestive system, circulatory system and nervous system diseases can be seen in humans as a result of consuming drinking water containing heavy metals in high concentrations. Aquatic sources contaminated with heavy metals cause toxic effects by accumulating in other living things besides humans. Heavy metal accumulation is observed in plants in agricultural areas irrigated with water containing heavy metals, and in animals grazing on such contaminated plants. Chatterjee and Chatterjee^8^ reported that growth reductions and phytotoxic effects occur as a result of changes in physiological and biochemical processes in plants grown in heavy metal contaminated areas. The protection of natural water resources and ensuring their continuous use for this purpose has become an increasingly important issue in Turkiye as well as in the rest of the world. For this reason, studies on water resources are especially important. In this study, metal pollution in the Pazarsuyu stream, which is an important water source, especially in agricultural applications, and the toxicity profile of this pollution were investigated.

Pazarsuyu is a stream located in the west of Bulancak district of Giresun province (Turkiye). Its length is 80 km, its catchment area is 874 km^2^, its annual flow is 674 hm^3^ and its flow is 21.4 m^3^/s. The stream takes its water source from the Giresun Mountains in the South. There are 18 regulator projects in total, including the Calıkobası, Oren, Gelen and Kovanlik hydroelectric power plants (HPPs) active on the stream. It is reported that the stream is exposed to domestic wastewater from Bozat, Aydindere and Kovanlik towns and most of this wastewater is not treated, directly or indirectly discharged into Pazarsuyu stream. In addition, the stream is exposed to intense pesticide pollution due to hazelnut cultivation in this region. For this reason, there are frequent reports in the media that cement mixes into the stream from the HPP constructions, fish deaths occur, the trees dry up, and the pollution in Pazarsuyu stream has received a great reaction^9,10^. Due to the increase in manufacturing industries and agricultural practices, Pazarsuyu is faced with various environmental problems. Studies investigating the pollution in Pazarsuyu stream are not yet at the desired level and there is no study examining the effects on organisms. In this study, both heavy metal pollution in the stream and the toxic effects of this pollution on *Allium cepa*, a bioindicator, were investigated. In addition, the effects of lycopene application as a solution in reducing toxic effects were investigated within the scope of the study.

In recent studies, extracts obtained from plants have been used to reduce the toxicity caused by heavy metals. Lycopene is a natural carotenoid synthesized by plants. In addition to giving color to fruits and vegetables, it absorbs light during photosynthesis and protects plants from stress conditions. Lycopene is an effective antioxidant that plays a role in scavenging free radicals. Due to the lipophilic nature of carotenoids, lycopene is mostly found in cell membranes and lipoprotein components. Lycopene exhibits the ability to disrupt the autooxidation of lipids by quenching lipid peroxyl radicals through electron transfer, hydrogen removal or addition^11^. In many literature studies, the protective property of lycopene has been associated with its antioxidant properties. Hedayati et al.^12^ reported that lycopene acts as a potential antioxidant, preventing heavy metal-induced toxicity by reducing oxidative stress. Çavuşoğlu et al.^13^ reported that administration of lycopene was protective against mercury-induced cytotoxicity and reduced genotoxic effects. The birth of life was with water, and it can also end with thirst. For this reason, scientists should draw attention to water pollution and keep it on the agenda by conducting studies on the control, protection and sustainability of water resources. There are many studies on natural water resources in the literature. In these studies, the pollution in the water source, the removal of pollution or its toxic effects are investigated. Existing studies on Pazarsuyu are mostly studies on the level of pollution. In this study, heavy metal pollution in stream water, the toxic effects of this pollution on a bio-indicator organism and the effects of lycopene application as a solution to reduce this toxicity were investigated. Toxic effects and protective properties were investigated in terms of physiological, biochemical, cytogenetic and anatomical aspects and a comprehensive profile was revealed.

## Materials and methods

### Research area and collection of samples

**Figure 1:**
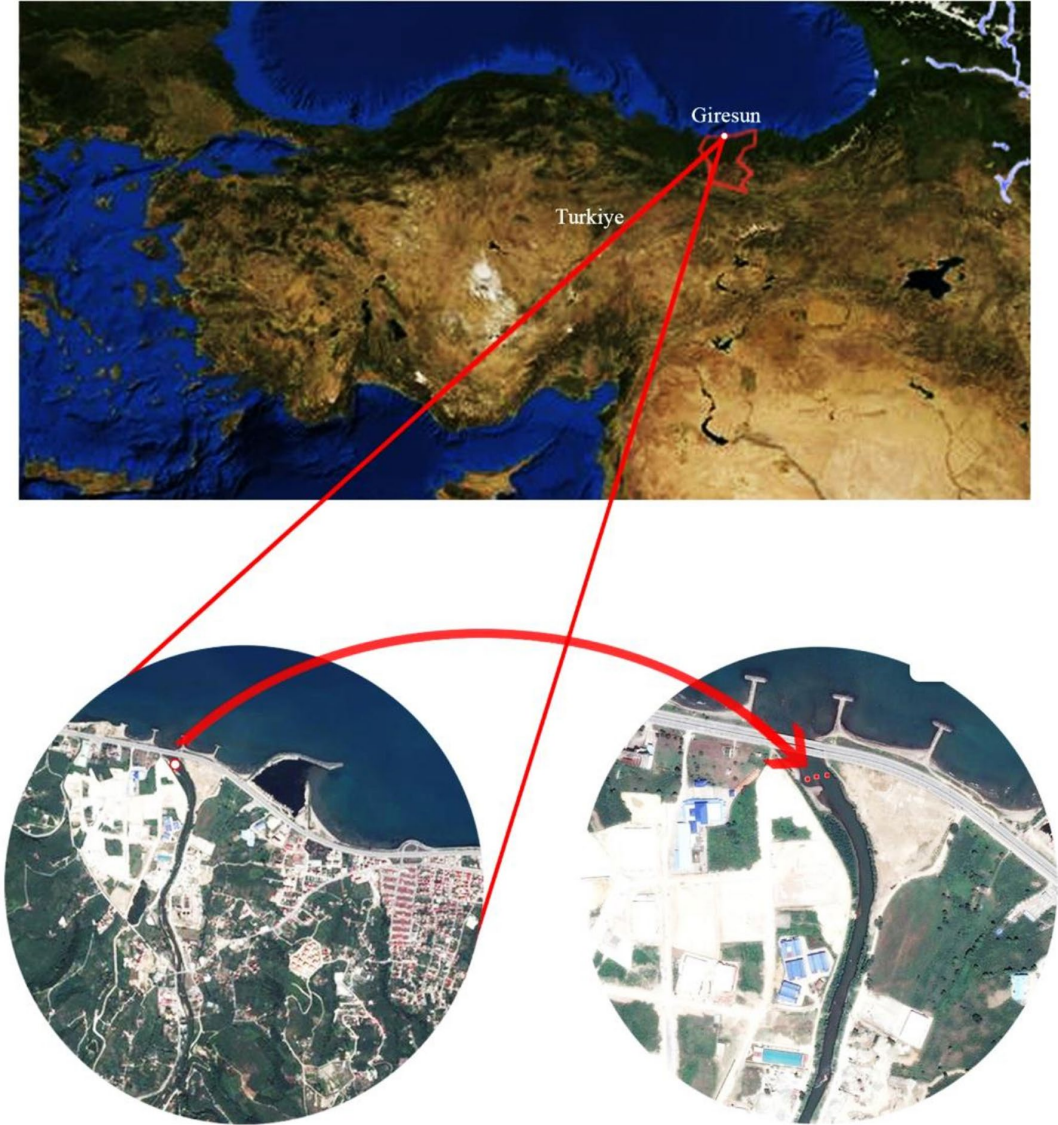
Sample collection station. The map was created using maps.yandex.com, 2021. Coordinates of the sample collection area (main area: 40° 56′ 39.0″ N, 38°10′ 29.5E) were entered into yandex maps and satellite images of the research area were obtained.

This study was carried out with samples collected from Pazarsuyu stream in Bulancak district of Giresun province in the Black Sea region. There are HPPs along the stream and hazelnut farming, which is the biggest income source of the district, is carried out around it. Water samples were collected on March 29, 2021. Water samples were taken from a distance of 100 m before Pazarsuyu stream reaches the Black Sea (Fig. 1). Samples were collected from different depths of the same spot into sterile 250 mL bottles. A total of 10 water samples were taken and heavy metal analysis was carried out.

### Heavy metal measurements

Collected water samples were filtered using a 0.45 µm membrane filter (Whatman Merck Millipore Corporation). Filtered water samples were diluted to 10 mL volume with 3% HNO_3_ for analysis. Heavy metal concentrations in the samples were measured using inductively coupled plasma-mass spectrometry (Bruker 820-MS ICP-MS). All measurements were performed in three replicates^14^. The recovery rates of metals in the reference material ranged between 90.7 and 104.4%.

### Test material and grouping principles

*A. cepa* bulbs of almost equal size were used as test material and lycopene (Sepe Natural-lycopene extract—90 capsules × 430 mg) was used as protective biological material. Bulbs are divided into 6 groups (Table 1).

**Table 1:** Experimental groups.

Group	Treatment
Group I	Control
Group II	215 mg/L lycopene
Group III	430 mg/L lycopene
Group IV	Stream water
Group V	Stream water + 215 mg/L lycopene
Group VI	Stream water + 430 mg/L lycopene

**Figure 2:**
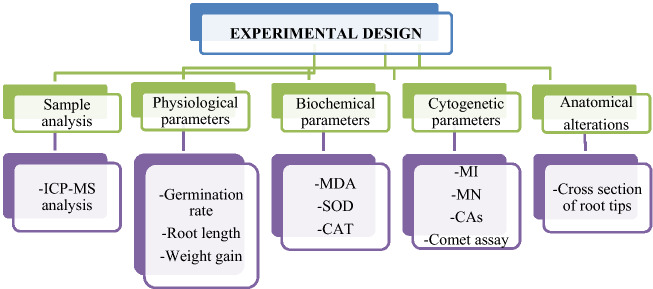
Experimental design of the study.

The bulbs in all groups were placed in pre-sterilized glass beakers with a diameter of 85 × 100 ml. The bulbs in the control group were germinated with tap water and the bulbs in the treatment group were germinated with stream water and 215 mg/L and 430 mg/L doses of lycopene. Germination was carried out at room temperature for 72 h. During the germination, daily controls of the bulbs and solution additions were made when necessary. At the end of 72 h, the bulbs were washed with distilled water and made ready for physiological, genetic, biochemical and anatomical analyzes^15^. In order to determine the toxicity of samples different parameters were used and the analyzed parameters are given in Fig. 2.

Experimental research on water and plant samples, including the collection of water samples and the supply of plant material, complies with institutional, national and international guidelines and legislation.

### Physiological parameter measurements

The effects of heavy metal ions in Pazarsuyu stream water and lycopene on *A. cepa* root growth were determined by measuring the root tips with a millimetric ruler, and their effects on weight gain were determined with measuring of the bulb weights with a precision balance before and after the application. The effects on the germination percentage were determined with the help of Eq. (1) ^16^.

1 $$ {\text{Germination}}\;{\text{percentage}}\left( \% \right) = \left[ {{\text{number}}\;{\text{of}}\;{\text{germinated}}\;{\text{seeds}}} \right]/\left[ {{\text{total}}\;{\text{number}}\;{\text{of}}\;{\text{seeds}}} \right] \times {1}00 $$

### Genotoxicity tests

In order to detect chromosomal abnormalities (CAs) and the presence of micronucleus (MN), the root tips of the germinating bulbs were cut to 1 cm, kept in Clarke solution (3 volumes of ethyl alcohol − 1 volume of glacial acetic acid) for 2 h, washed in ethyl alcohol (96%) for 15 min and stored in the refrigerator at + 4 °C in ethyl alcohol (%70). For permanent preparation, root tips were hydrolyzed in 1 N hydrochloric acid for 17 min in an oven at 60 °C and kept in acetic acid (45%) for 30 min at the end of the period. Afterwards, root tips were stained with acetocarmine for 24 h, crushed with acetic acid (45%) and examined under IRMECO IM-450 TI model research microscope, and the presence of CAs and MN were photographed at × 500 magnification^17^. The criteria proposed by Fenech et al.^18^ were used to determine the presence of MN. According to these criteria, for a formation to be MN; diameter should be 1/3 of the nuclear diameter, round or oval in shape and clearly distinguishable from the nucleus. Mitotic index (MI), which shows the ratio of cells entering mitosis to total cells, was calculated with the help of Eq. (2).

2 $$ {\text{MI}} = \left[ {{\text{number}}\;{\text{of}}\;{\text{cells}}\;{\text{undergoing}}\;{\text{mitosis}}} \right]/\left[ {{\text{total}}\;{\text{number}}\;{\text{of}}\;{\text{cells}}} \right] \times {1}00 $$

### Comet assay (single-cell gel electrophoresis)

The protocol proposed by Chakraborty et al.^19^ was applied for alkaline single-cell gel electrophoresis. Comets were analyzed with Comet assay software (CASP) version 1.2.3b^20^ with the parameters of tail DNA length. A total of 2000 cells were analyzed for each group, 200 in each bulb for DNA damage. The extent of DNA damage was scored from 0 to 4 depending upon the level of DNA damage. The cells were classified into five categories based on tail DNA length ranging from zero to four according to Collins^21^. The total DNA damage per group, expressed as arbitrary units, was calculated using Eq. (3).

3 $$ Arbitrary\;unit = \sum\limits_{{i = 0}}^{4} {Nixi}  $$

(i: degree of damage (0, 1, 2, 3, 4), Ni: the number of cells in i degree).

### Biochemical parameter measurements

#### Malondialdehyde measurements

MDA measurements were carried out according to the method proposed by Unyayar et al.^22^. 0.5 g of root tip was homogenized in 1 mL of trichloroacetic acid (TCA-5%) solution. The homogenate was transferred to a new tube and centrifuged for 10 min at 12,000*g* at 24 °C. Equal volumes of thiobarbituric acid (TBA-0.5%) and supernatant were transferred to a new tube, incubated in TCA solution (20%) at 96 °C for 30 min, then placed in an ice bath and centrifuged at 10,000*g* for 5 min. The absorbance of the supernatant was measured at 532 nm and the MDA level was expressed as μM/g FW.

#### Enzyme measurements

Enzyme extraction was carried out at 4 °C on a sample of 0.5 g of fresh root tips, washed with distilled water and homogenized in 5 mL of NaH_2_PO_4_ buffer (50 mM, pH 7.8). The mixture was centrifuged at 10,500 g for 20 min and the supernatant was used for enzyme analysis^23^.

#### SOD measurement

The measurement of the SOD activity was performed according to the method proposed by Beauchamp and Fridovich^24^. The reaction solution was prepared using 1.5 mL NaH_2_PO_4_ buffer, 0.3 mL C_5_H_11_NO_2_S, 0.3 mL nitroblue tetrazolium chloride, 0.3 mL EDTA-Na_2_, 0.3 mL C_17_H_2_ON_4_O_6_, 0.01 mL enzyme extract, 0.01 mL insoluble polyvinylpyrrolidone and 0.28 mL de-ionized water. The reaction was initiated by placing the tubes under 15 W fluorescent lamps for 10 min and ended by keeping the tubes in the dark for 10 min. The absorbance was read at 560 nm and the SOD activity was expressed as U/mg FW^23^.

#### CAT measurement

The measurement of the CAT activity was carried out according to the method proposed by Beers and Sizer^25^. CAT activity was measured in 2.8 mL of reaction solution containing 0.3 mL of 0.1 M hydrogen peroxide, 1.0 mL of distilled water and 1.5 mL of 200 mM NaH_2_PO_4_ buffer by using a UV–VIS spectrophotometer. The reaction was initiated by adding 0.2 mL of enzyme extract; CAT activity was measured by monitoring the decrease in absorbance at 240 nm as a result of hydrogen peroxide consumption and expressed as OD_240nm_ min/g^23^.

### Determination of meristematic cell damages

The root tips were washed with distilled water and subsequently placed in foam material and their cross sections were taken with the help of a sharp razor blade. The sections were stained with methylene blue (5%) for 2 min. Anatomical damages and changes were investigated in cross sections by using IRMECO IM-450 TI model research microscope and photographed at × 200 magnification^26^.

### Recovery effects of lycopene

In order to determine the recovery effects (RE) of lycopene, the data of the lycopene applied groups, the data of the stream water application group and the data of the control group were used. RE of lycopene was calculated using the following Eq. (4).

4 $$ {\text{Recovery}}\;{\text{effect}}\;\% = \left[ {\left( {{\text{D}}_{{1}} - {\text{D}}_{{2}} } \right)/\left( {{\text{D}}_{{3}} - {\text{D}}_{{2}} } \right)} \right] \times {1}00 $$

D_1_: data of 215 mg/L lycopene or 430 mg/L lycopene applied group, D_2_: data of stream water treated group, D_3_: data of control group.

### Statistical analysis

The data obtained were evaluated using the SPSS Statistics 22 (IBM SPSS, Turkiye) package program. Data are shown as mean standard deviation (SD). The statistical significance between the means was determined with the help of one-way analysis of variance (One-way ANOVA) and Duncan tests, and it was considered statistically significant when the determined p value was less than 0.05.

In addition, Pearson correlation analysis (two-sided) was performed in RStudio and correlation plots were performed with the corrplot package^27^. Principal component analysis (PCA) was performed for physiological, genetic and biochemical parameters, which are different biomarkers of toxicity for each dose tested. The FactoMineR^28^ and factoextra^29^ packages in RStudio were used to perform principal component analysis (PCA)^30^.

## Result and discussion

### Heavy metal concentrations

Heavy metal concentrations in water samples collected from Pazarsuyu stream are shown in Table 2. As a result of the analysis, Fe was detected in high concentrations in water samples, while the presence of Cd metal in the lowest concentration was detected. Among these metals, Sr, Ba, Be, Mo, Li, Te and Ti are not among the elements whose limit values are given in the "Regulation on Water Intended for Human Consumption" published by the Ministry of Health of the Republic of Turkiye. The concentrations of other heavy metals are in accordance with the values specified in the regulation. The main causes of this heavy metal pollution in Pazarsuyu stream are pesticides originating from hazelnut cultivation in agricultural areas around the stream, power stations established on the stream and industrial establishments located near the stream^31^. In the literature, there are studies on the pollution of water by heavy metal ions as a result of the activities of industrial establishments located near natural water resources. Vitek et al.^32^ investigated the extent of heavy metal pollution in the aquatic ecosystem of the Loučka River in the Czech Republic and reported that the heavy metals Ni and Cr were highest in river water. In the literature, heavy metal pollution in different water resources in the world has been investigated and important results have been obtained. Moore et al.^33^ investigated the heavy metal pollution in the water and sediment samples of the streams in Sungun region and observed that the Cu, Mo, Pb, Zn and Ni concentrations exceeded the maximum concentrations allowed in the international legislation. Wasiu et al.^34^ detected Cu, Zn, Fe, Cd, Pb and As metals in the rivers in the Osun State, Nigeria and reported that their presence rates were above the limits set by WHO and Nigeria drinking water quality standards.

**Table 2:** Heavy metal concentrations measured in stream water (µg/L).

Element	Concentration (µg/L)	Parametric value for potable and utility water (µg/L)	Recovery (%)
Iron (Fe)	126.3 ± 33.8	200	95.5
Strontium (Sr)	62.5 ± 2.03	–	100.9
Barium (Ba)	21.2 ± 2.17	–	96.1
Beryllium (Be)	9.35 ± 0.38	–	95.9
Molybdenum (Mo)	8.61 ± 2.51	–	104.2
Lithium (Li)	1.96 ± 0.85	–	93.2
Nickel (Ni)	1.19 ± 0.68	20	104.4
Aluminum (Al)	0.784 ± 0.97	200	90.7
Tellurium (Te)	0.502 ± 0.45	–	94.8
Copper (Cu)	0.453 ± 0.44	2	104.8
Titanium (Ti)	0.098 ± 0.07	–	91.6
Cadmium (Cd)	0.028 ± 0.07	5	98.3

Data are shown as mean ± standard deviation (SD). The parametric value expresses the monomer residue concentration in the water originating from the polymer in contact with the water.

### Physiological parameters

Physiological changes in *A. cepa* caused by heavy metal pollution in Pazarsuyu stream are shown in Table 3. The highest germination, root length and weight gain were measured in control group (Group I) and Group II and Group III, which were exposed to two different doses of lycopene. No statistical difference was observed in terms of germination percentage, root length and weight gain in the control group and only lycopene applied groups (p > 0.05). This result shows that lycopene application alone did not cause any abnormality in germination-related parameters. Stream water application caused a decrease in all investigated physiological parameters. Compared to Group I (control), the germination percentage decreased by half (50%), root length approximately 3.64 times (73%) and weight approximately 3.06 times (68%) in Group IV treated with stream water. These decreases were found to be statistically significant (p < 0.05). In Group V and Group VI, in which stream water and lycopene were applied together, lycopene decreased the effects of heavy metals in the stream water and again caused statistically significant (p < 0.05) increases in the investigated physiological parameter values. It was observed that these observed increases were more pronounced at the 430 mg/L dose of lycopene. Compared to Group IV treated with stream water, the percentage of germination increased by 25%, root length by 2 times (100%) and weight by approximately 1.97 times (98%) in Group VI treated with 430 mg/L dose of lycopene.

**Table 3:**
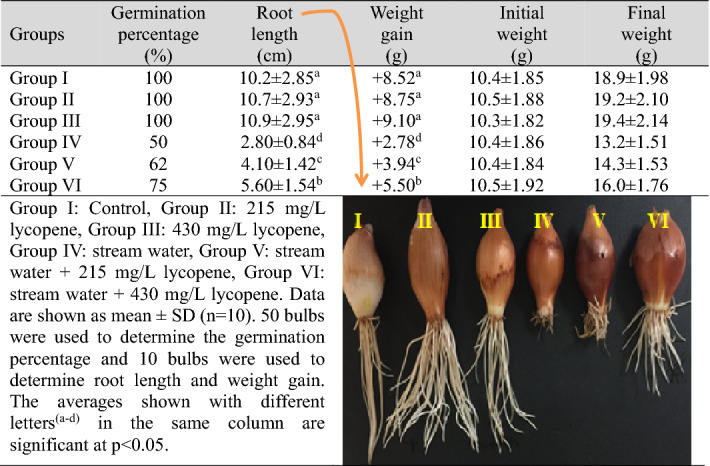
The effects of stream water and lycopene application on germination parameters.

The fact that stream water application causes a decrease in germination-related parameters in *A. cepa* may be associated with heavy metal pollution. The decreases observed in root elongation and weight gain in the stream water applied group can be explained by the reduction of water and nutrient uptake from the roots by heavy metal ions. It has been reported in the literature that in the presence of heavy metals, water and mineral uptake by the roots is inhibited, photosynthesis and nitrogen metabolism are impaired, and root and shoot growth are reduced. The production of reactive oxygen species (ROS) has been shown as the main cause of these negative effects caused by heavy metal toxicity in plants^35^. Similarly, Doğan et al.^36^ determined that heavy metal pollution in Civil (Ordu-Turkey) stream water caused oxidative stress in *A. cepa*, resulting in significant reductions in germination percentage, root length and weight gain. In another study, it was determined that Melet river (Ordu, Turkey) water samples containing heavy metals such as Pb, Al, Ni, Cr and Fe limited the growth and development of *V. faba*^37^.

### Genotoxicity

**Figure 3:**
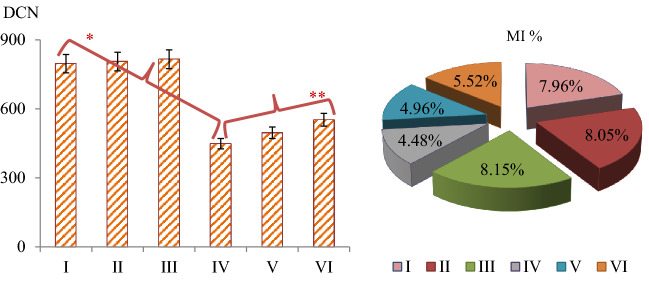
Effects of stream water and lycopene treatments on dividing cell number (DCN) and mitotic index (MI). MI was calculated by counting 10.000 cells in each group. *Indicates the statistical difference between Groups I and IV, **indicates statistical difference between Groups IV and VI (p < 0.05).

**Figure 4:**
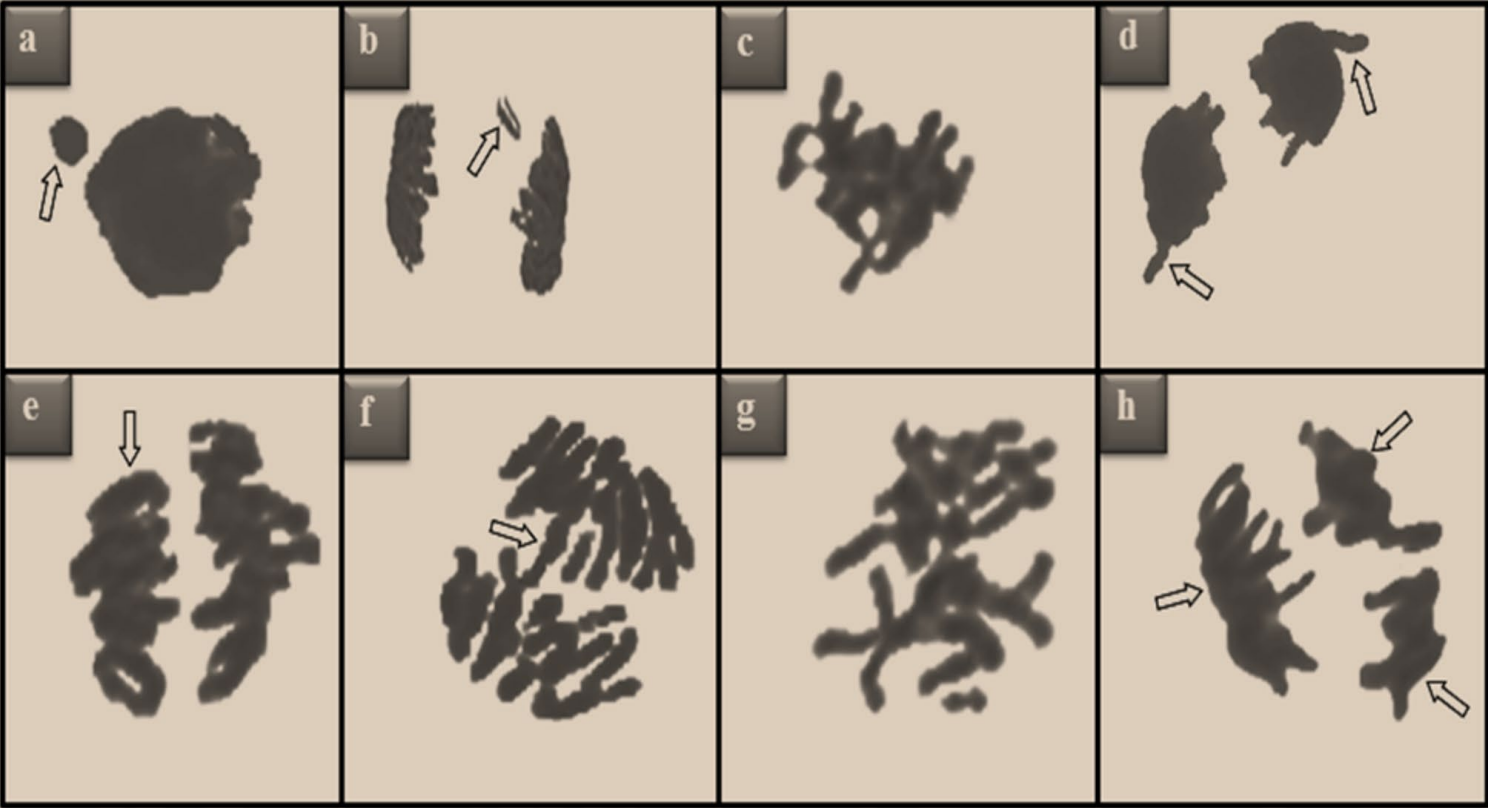
CAs induced by heavy metal ions in stream water. MN (**a**), fragment (**b**), sticky chromosome (**c**), vagrant chromosome (**d**), unequal distribution of chromatin (**e**), bridge (**f**), disordered mitosis (**g**), multipolar anaphase (**h**).

The genotoxic effects of stream water and the protective properties of lycopene were determined by investigating the MI ratios and MN and CAs frequencies. The effects of stream water on dividing cell numbers and MI are shown in Fig. 3. Similar MI rates were determined in the control and only-lycopene treated groups. The number of dividing cells in Group IV treated with stream water decreased by 43.6% compared to the control group. Stream water application promoted genotoxicity in root tip cells, causing an increase in MN and CAs frequencies (Table 4). While no MN formation was observed in the control and only lycopene applied groups, MN was determined with a frequency of 74.1 ± 7.12 in the stream water applied group. The increase in MN frequency was statistically significant (p < 0.05). Also, stream water application induced CAs such as fragment, sticky chromosome, vagrant chromosome, unequal distribution of chromatin, bridge, irregular mitosis and multipolar anaphase (Fig. 4). The greatest effect of heavy metals in stream water on chromosomes was observed in the form of fragment formation. The application of lycopene together with stream water reduced the effect of heavy metals in the water and caused an improvement in the investigated genotoxicity indicators. This improvement was observed to be more pronounced at the 430 mg/L dose of lycopene. Compared to Group IV, the number of MNs decreased approximately 1.60 times (38%) and MI increased approximately 1.23 times (1.04%) in Group VI, where a dose of 430 mg/L of lycopene was administered together with stream water.

**Table 4:** Protective role of lycopene against genotoxicity induced by heavy metal ions in stream water.

Parameters	Group I	Group II	Group III	Group IV	Group V	Group VI
MN	0.00 ± 0.00^d^	0.00 ± 0.00^d^	0.00 ± 0.00^d^	74.1 ± 7.12^a^	60.3 ± 6.18^b^	46.4 ± 4.76^c^
FRG	0.00 ± 0.00^d^	0.00 ± 0.00^d^	0.00 ± 0.00^d^	58.4 ± 5.12^a^	46.5 ± 4.78^b^	35.9 ± 4.11^c^
SC	0.80 ± 0.64^d^	0.50 ± 0.36^d^	0.60 ± 0.44^d^	42.9 ± 4.24^a^	35.7 ± 3.66^b^	26.5 ± 2.93^c^
VC	0.00 ± 0.00^d^	0.00 ± 0.00^d^	0.00 ± 0.00^d^	30.4 ± 3.12^a^	23.8 ± 2.84^b^	17.4 ± 2.15^c^
UDC	0.30 ± 0.24^d^	0.00 ± 0.00^d^	0.00 ± 0.00^d^	24.6 ± 1.98^a^	18.7 ± 1.53^b^	12.6 ± 1.28^c^
B	0.00 ± 0.00^d^	0.00 ± 0.00^d^	0.00 ± 0.00^d^	19.7 ± 1.85^a^	12.4 ± 1.55^b^	7.70 ± 1.12^c^
IM	0.00 ± 0.00^d^	0.00 ± 0.00^d^	0.00 ± 0.00^d^	11.8 ± 1.14^a^	7.10 ± 0.92^b^	3.20 ± 0.64^c^
MA	0.00 ± 0.00^c^	0.00 ± 0.00^c^	0.00 ± 0.00^c^	5.40 ± 0.84^a^	2.50 ± 0.62^b^	0.50 ± 0.44^c^

Group I: Control, Group II: 215 mg/L lycopene, Group III: 430 mg/L lycopene, Group IV: stream water, Group V: stream water + 215 mg/L lycopene, Group VI: stream water + 430 mg/L lycopene. Data are shown as mean ± SD (n = 10). MN and CAs were calculated by counting 1,000 cells in each group. The averages shown with different letters^(a–d)^ in the same line are significant at p < 0.05.

*MN* micronucleus, *FRG* fragment, *SC* sticky chromosome, *VC* vagrant chromosome, *UDC* unequal distribution of chromatin, *B* bridge, *IM* irregular mitosis, *MA* multipolar anaphase.

The main reason for the genotoxicity induced by stream water can be explained by the direct or indirect interaction of heavy metals in the stream water with the chromosomes of the root tip cells. It has been reported in the literature that heavy metals cause serious damage to DNA by reacting with DNA (directly) or by producing ROS (indirectly). It has also been reported that heavy metal exposure causes disruptions in genome integrity. Inhibition in repair mechanisms of DNA, double-strand breaks, MN, breaks, sister chromatid changes and variations in genes are important damages caused by heavy metals in chromosomes^38^. The genotoxic effects of stream waters containing heavy metal pollution are also reported in some studies. Türkmen et al.^39^ reported significant increases in CAs and MN frequency and significant decreases in MI in *A. cepa* root tip cells exposed to Melet (Ordu-Turkey) stream water polluted with Pb, Fe, Al, Ni, Cu, Zn, Cr and Cd ions. Doğan et al.^36^ reported that heavy metal-containing stream water application reduces cell proliferation and causes genotoxic effects in *A. cepa.*

### Comet assay

**Figure 5:**
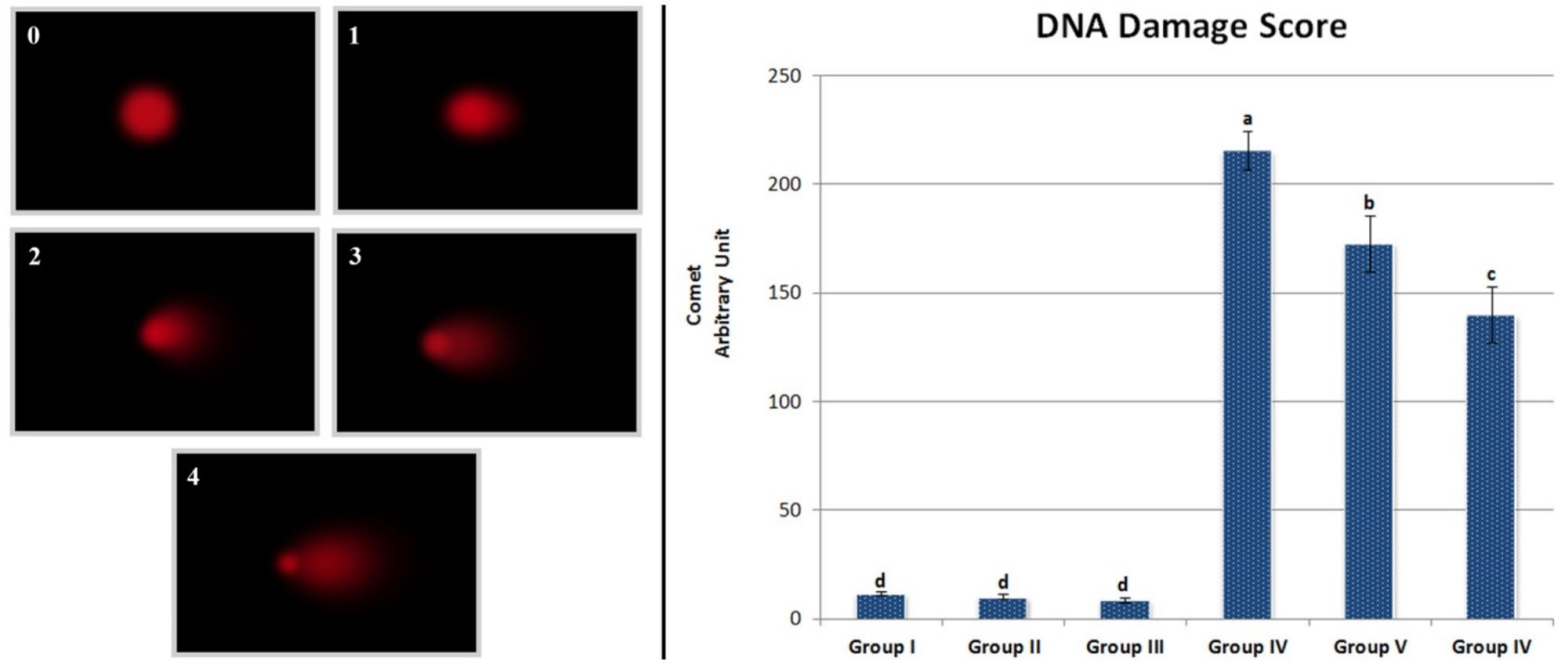
Effects of stream water and lycopene application on the DNA of *A. cepa* root meristem cells. Group I: control, Group II: 215 mg/L lycopene, Group III: 430 mg/L lycopene, Group IV: stream water, Group V: stream water + 215 mg/L lycopene, Group VI: stream water + 430 mg/L lycopene. 0: no damage, 1: low damage, 2: moderate damage, 3: high damage, 4: extreme damage. Data are given as mean ± SEM. Means shown with different letters^(a–d)^ in the graph are significant at p < 0.05.

The genotoxicity caused by stream water polluted with heavy metal was also supported by the "Comet assay" technique used to detect DNA damage and amount at the cell level. In this context, the effects of stream water and lycopene treatments on DNA in *A. cepa* root tip cells are shown in Fig. 5. Findings from Groups II and III showed that lycopene administration alone did not make a significant difference in DNA damage compared to the control (p > 0.05). However, exposure to stream water caused statistically significant (p < 0.05) DNA damage in *A. cepa* root tip cells. While the mean DNA damage score was 11.33 ± 0.72 in the control group (Group I), there was a sharp increase in Group IV with stream water alone and the mean DNA damage score was 215.17 ± 8.91. In Group V, where stream water and 215 mg/L lycopene were given together, the DNA damage score decreased to 172.67 ± 12.89. In Group VI, where 430 mg/L lycopene was given together with stream water, the DNA damage score decreased further to 139.67 ± 13.03. The increase in DNA damage score due to stream water exposure indicates that heavy metal ions in stream water induce DNA double-strand breaks in *A. cepa* root tip cells. On the other hand, it was determined that there was a statistically significant (p < 0.05) difference between the DNA damage scores determined in Group IV exposed to stream water and in groups (Group V and VI) treated with lycopene together with stream water. The Comet test is a reliable test used to detect DNA single-strand breaks in all cell types. In the Comet test, damaged DNA displays a "comet" structure on electrophoresis, and the percentage of DNA in the tail is directly proportional to the damage^40^. The high DNA damage score in the stream water applied group indicates DNA fragmentation and DNA chain breaks. These damages also confirm the genotoxic effect detected by MN and CA tests. Similarly, Scalon et al.^41^ investigated the genotoxic effect of heavy metal-contaminated stream water application as a result of industrial discharges with the comet test in *Hyphessobrycon luetkenii* (Boulenger) and reported that water samples containing heavy metals had a genotoxic effect by causing DNA fragmentation.

### Biochemical parameters

The effects of heavy metal-contaminated stream water on biochemical parameters are shown in Table 5. There was no statistically significant (p > 0.05) difference between the control group and the lycopene-only groups in terms of the tested parameters. Significant changes were observed in the levels of MDA, SOD and CAT in the stream water applied group. Compared to the control group, MDA level increased by approximately 2.90-fold, SOD activity by 2.84-fold and CAT activity by 2.68-fold in Group IV treated with stream water. Co-administration of stream water and lycopene reduced the effects of heavy metals and caused a decrease in the levels of the investigated biochemical parameters. It was observed that this decrease was more pronounced at the 430 mg/L lycopene dose. In Group VI treated with 430 mg/L lycopene MDA level decreased approximately 1.82-fold, SOD activity 1.43-fold and CAT activity 1.39-fold compared to Group IV treated with stream water.

**Table 5:** Effect of stream water and lycopene application on selected biochemical parameters.

Groups	MDA (µM/g FW)	SOD (U/mg FW)	CAT (OD_240 nm_ min/g FW)
Group I	10.5 ± 3.75^d^	105.6 ± 7.16^d^	1.10 ± 0.68^d^
Group II	10.2 ± 3.68^d^	104.8 ± 6.84^d^	1.03 ± 0.62^d^
Group III	9.90 ± 3.52^d^	102.2 ± 6.55^d^	0.960 ± 0.57^d^
Group IV	30.4 ± 4.78^a^	300.4 ± 14.85^a^	2.95 ± 0.98^a^
Group V	23.6 ± 3.86^b^	270.1 ± 12.88^b^	2.50 ± 0.83^b^
Group VI	16.7 ± 3.15^c^	210.3 ± 10.54^c^	2.12 ± 0.74^c^

Group I: Control, Group II: 215 mg/L lycopene, Group III: 430 mg/L lycopene, Group IV: stream water, Group V: stream water + 215 mg/L lycopene, Group VI: stream water + 430 mg/L lycopene. Data are shown as mean ± SD (n = 10). The averages shown with different letters^(a–d)^ in the same column are significant at p < 0.05.

Increases in MDA, SOD and CAT levels in root tip cells indicate that oxidative stress is induced in the cell. Especially considering that MDA is a peroxidation product, it can be said that stream water application causes lipid peroxidation. MDA is a well-known product of lipid peroxidation and is used as a marker of cell membrane damage. Increasing oxidative stress in the cell causes peroxidation of cell membrane lipids and MDA level in the cell increases^42^. The increase in MDA levels in the roots of *A. cepa* exposed to river water in Group IV can be explained by the oxidative stress induced by heavy metals in the water and the damage to the cell membrane. Increased oxidative stress in cells is neutralized by endogenous antioxidants. SOD and CAT are antioxidant enzymes that suppress the formation of free radicals in cells or reduce their effects. While SOD neutralizes the superoxide radical in the cell, CAT acts on hydrogen peroxide and catalyzes its conversion to harmless molecules such as water and oxygen^35^. When evaluated in this context, the increase in SOD and CAT levels in stem cells exposed to river water can be explained by the fact that heavy metals in water promote oxidative stress in the cell and increase the SOD and CAT levels of the cell. These results are also consistent with the results of some studies investigating the biochemical toxicity induced by heavy metals in water. Türkmen et al.^39^ reported that stream water contaminated with petroleum wastewater and containing heavy metals increased root MDA levels in *A. cepa* test material and caused oxidative damage. Aljahdali and Alhassan^43^ stated that sediment and water samples containing heavy metal pollution above the limit values significantly increased CAT, SOD and glutathione S-transferase enzyme activities in *C. serrulata.*

### Anatomical observations

**Figure 6:**
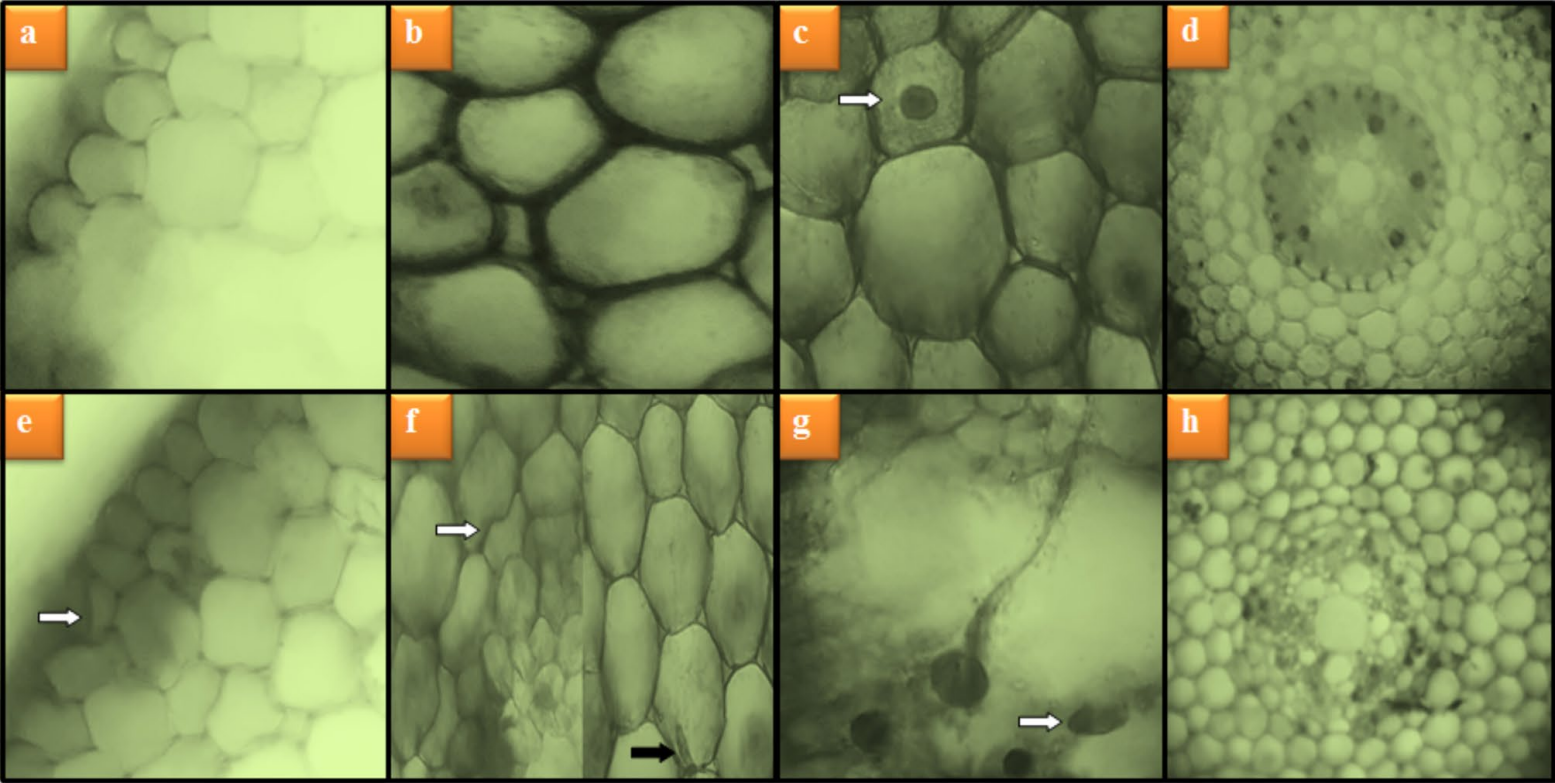
Meristematic cell damages caused by heavy metals in stream water. Normal appearance of epidermis cells (**a**), normal appearance of cortex cells (**b**), normal appearance of cell nucleus-*oval* (**c**), normal appearance of vascular tissue (**d**), epidermis cell damage (**e**), cortex cell damage-*white arrow*, accumulation of some substances in cortex cells-*black arrow* (**f**), flattened cell nucleus (**g**), unclear vascular tissue (**h**).

Anatomical damages caused by heavy metals in stream water are shown in Fig. 6. No damage was observed in the root tip meristem cells of the control group, Group II and III treated with only-lycopene. Stream water application in Group IV induced anatomical damages such as epidermis and cortex cell damage, accumulation of some substances in cortex cells, flattened cell nucleus and unclear appearance of conduction tissue in root tip meristem cells. The application of lycopene together with stream water decreased the negative effects of heavy metals on root cells and caused a decrease in the severity of damage to meristem cells (Table 6). It was determined that these decreases were more pronounced at the 430 mg/L dose of lycopene.

**Table 6:** Protective role of lycopene against meristematic cell damage induced by heavy metal ions in stream water.

Groups	ECD	CCD	SACC	FCN	UVT
Group I	−	−	−	−	−
Group II	−	−	−	−	−
Group II	−	−	−	−	−
Group IV	+++	+++	++	+++	++
Group V	++	++	+	++	+
Group VI	+	+	−	+	−

Group I: Control, Group II: 215 mg/L lycopene, Group III: 430 mg/L lycopene, Group IV: stream water, Group V: stream water + 215 mg/L lycopene, Group VI: stream water + 430 mg/L lycopene. ECD: epidermis cell damage, CCD: cortex cell damage, SACC: substance accumulation in cortex cells, FCN: flattened cell nucleus, UVT: unclear vascular tissue. (−): no damage, (+): little damage, (++): moderate damage, (+++): severe damage.

It is thought that these anatomical damages, especially epidermis-cortex cell damage and flattened cell nucleus damage caused by heavy metals occur as a result of the defense mechanisms developed by the plant against heavy metals. Because, in microscopic examinations, it was observed that roots exposed to heavy metals in stream water increased the number and order of epidermis and cortex cells in order not to take heavy metals into the cells. These changes can increase the contact of the epidermis and cortex cells with each other, causing their suppression and therefore deformities in the shapes and nucleus of the cells. The information included in the literature supports our opinion that plants exposed to heavy metals can develop various physical and chemical defense mechanisms, such as accumulating, storing and crystallizing metals, forming structural changes in the cell membrane and wall, increasing the number of vacuoles and metal-binding protein synthesis^44^. Similar studies in the literature support our findings. Doğan et al.^36^ observed that Civil (Ordu-Turkiye) stream water polluted with Cr, Co, Ni, Cu, Cd, Pb ve Fe heavy metals caused anatomical damages such as necrosis, thickening of the cortex cell wall, cell deformation, accumulation of some substances in the cortex cells, flattened cell nucleus and unclear vascular tissue in *A. cepa* root tip meristem cells.

### Correlation and principal component analysis of parameters

**Figure 7:**
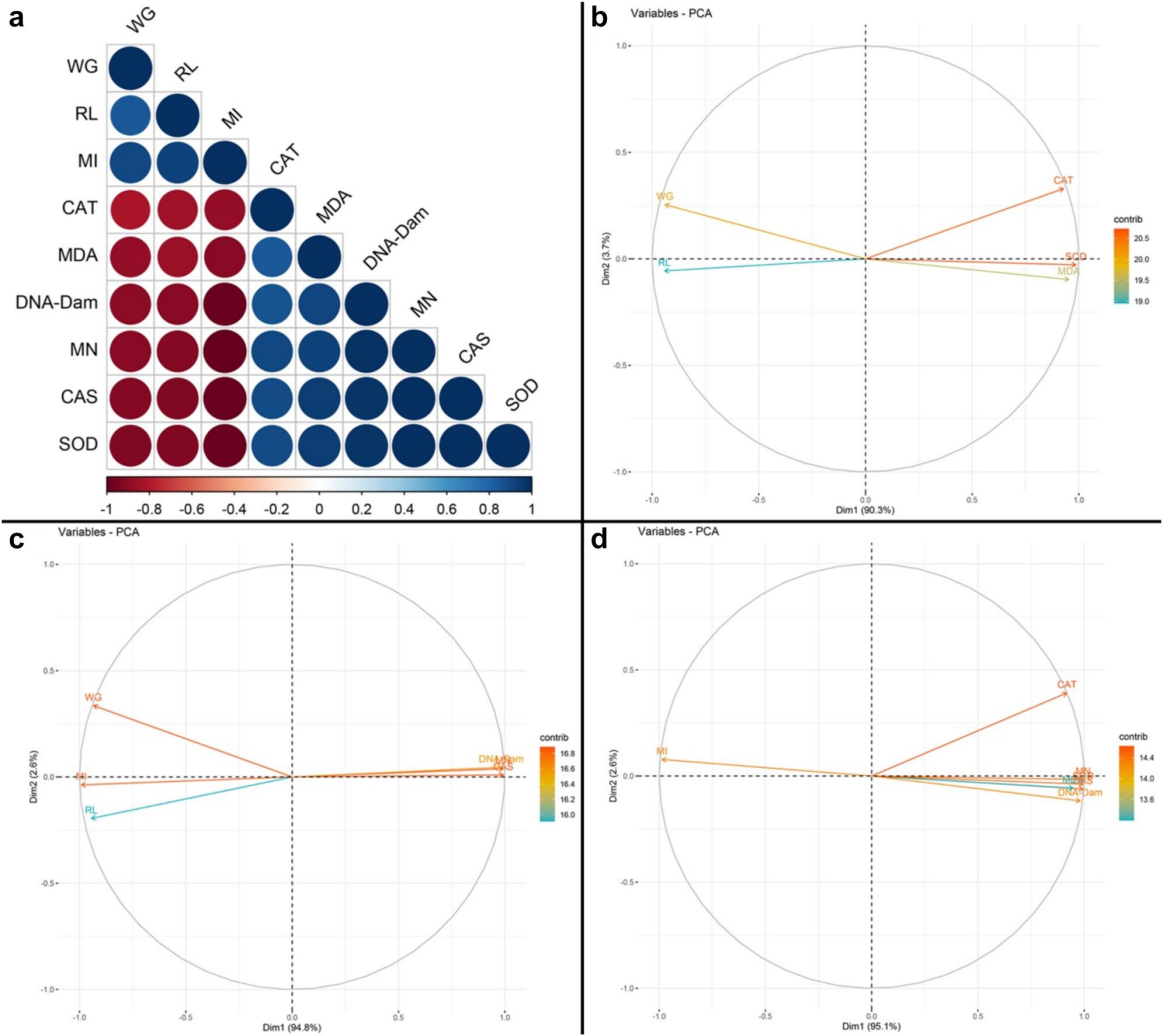
Correlation and principal component analyses (PCA) of physiological, biochemical and genetic response parameters. Correlation of physiological, biochemical and genetic parameters (**a**), PCA analysis of physiological parameters and biochemical parameters (**b**), PCA analysis of physiological parameters and genetic parameters (**c**), PCA analyses of genetic and biochemistry parameters (**d**). *WG* weight gain, *RL* root length, *DNA-Dam* DNA damage. Pearson correlation analysis (two-sided) was performed and visualized with Rstudio software. Positive correlations are shown in blue and negative correlations in red. The color intensity and the size of the circle are proportional to the correlation coefficients.

The correlation analysis of all parameters is shown in Fig. 7a. Positive correlations are shown in blue and negative correlations are shown in red. Color intensity and circle size are proportional to correlation coefficients. Weight gain, root length, MI parameters showed negative correlations with CAT and SOD activities, MDA level, DNA damage score, MN formation frequency and CAs formations. The obtained correlation data showed that the investigated parameters had significant positive or negative effects on each other. The overall physiological, biochemical, and genetic effects of stream water and lycopene administrations, as well as clustering among biomarkers after the application time, were visualized using principal component analysis (PCA) and are given in Fig. 7b–d. In Fig. 7b, which deals with PCA analyzes of physiological parameters and biochemical parameters, the first two dimensions of the biplot explained 94.0% of the overall variance, with the first axis (dim1) distinguishing control and application groups clearly (90.3%). The dim2 as a visualization aid accounted for 3.7% of the overall variance. As a result of the analysis, it was found that MDA level, SOD and CAT activities were close to each other, with a very positive component on dim1 axis, and weight gain and root length parameters are very negative component on dim1 axis. PCA analyzes of physiological parameters and genetic parameters are given in Fig. 7c. In the biplot, the first two dimensions, the first axis (dim1) 94.8% and the second axis (dim2) 2.6%, explain 97.4% of the overall variance. As a result of the analysis, it was found that DNA damage score, MN and CAs formation levels were close to each other, with a very positive component on dim1 axis and a slight positive on dim2 axis. Figure 7d shows PCA analyses of genetic and biochemistry parameters. The first two dimensions in the biplot, the first axis (dim1) 95.1% and the second axis (dim2) 2.6%, explain 97.7% total variation. As an outcome of the assessment, it was found that MN frequency, DNA damage score, CAs formation, MDA level, CAT and SOD activities were very positive components on dim1 axis. All these PCA analyzes confirm the interrelationships of all investigated physiological, genetic and biochemical parameters.

### Recovery effects of lycopene

**Figure 8:**
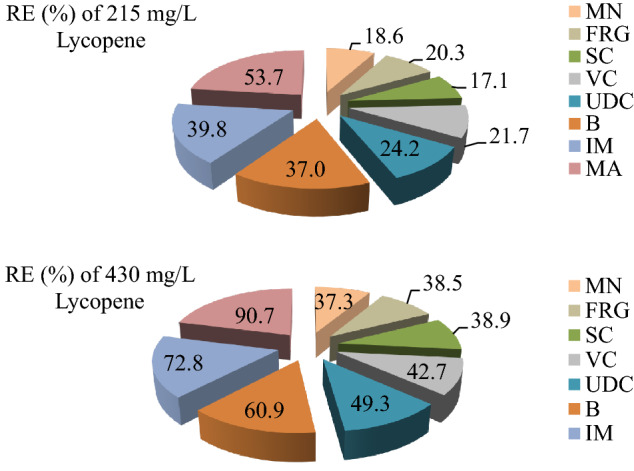
Recovery effects of lycopene doses against MN and CAs frequencies.

Stream water treatment induced the formation of MN and CAs in *A. cepa* root tip cells, and the lycopene application showed a protective role by reducing the frequencies of these abnormalities (Fig. 8). The protective role of lycopene against the genotoxic effect increased depending on the dose. Administration of 215 mg/L lycopene provided protection against MN and CAs in the range of 17.1–53.7%. In the application of 430 mg/L lycopene, a protection between 37.3 and 90.7% was obtained. The highest protection was observed against multipolar anaphase formations at both doses.

In recent years, plant extracts such as carotene, grape seed, *Ginkgo biloba* L., green coffee and green tea have been used to reduce the toxicity caused by environmental pollutants such as heavy metal ions and pesticides. In this study, lycopene, a powerful antioxidant, was used in order to reduce the effects of physiological, genetic, biochemical and anatomical toxicity caused by heavy metal pollution in Pazarsuyu stream. Due to its antioxidant properties, lycopene reduced the toxic effects of heavy metal ions in stream water and improved physiological, genetic, biochemical and anatomical parameters. This curative role of lycopene has been associated with neutralizing oxidative stress induced by heavy metals in stream water. Lycopene protects the cell against oxidative stress by neutralizing free radicals through different mechanisms such as adduct formation, electron transfer to the radical, and allylic hydrogen abstraction. And also lycopene has a scavenging effect on singlet oxygen and peroxyl radicals formed by toxic agents in the cell^45^. The protective feature of lycopene against the multi-toxic effects caused by Pazarsuyu stream water containing heavy metals is due to these activities. The protective feature of lycopene, which we detected in this study, has also been demonstrated by many studies. Çavuşoğlu et al.^46^ reported that lycopene provides significant protection against physiological, biochemical and genetic toxicity induced by Pb, Zn, Fe, Cu, Ni, Cd and Hg. Kalefetoğlu Macar et al.^47^ reported that lycopene provides dose-dependent protection against genotoxic and anatomical damage caused by Co heavy metal ion at a dose of 5.5 mg/L in *A. cepa* roots.

## Conclusion

The Pazarsuyu Stream, which is located in the Bulancak district of Giresun province and reaches the Black Sea, is an important water source in the region and is used for many purposes, especially for agricultural applications. In this study, it was determined that there is heavy metal pollution in Pazarsuyu stream above the limit values specified in national and international legislation. The extent of water pollution was not only determined by measurement-based methods, but also the biological effects of pollution in *A. cepa*, which is the indicator test material, were investigated in all aspects. The water samples collected from the stream caused toxicity by promoting changes in the physiological, genetic, biochemical traits as well as in anatomical structure of the *A. cepa* test material. The application of lycopene together with stream water reduced the deleterious effects of heavy metals and again caused a significant improvement in all investigated parameters. It was determined that this improvement was even more pronounced when 430 mg/L dose of lycopene was applied. Misuse of water resources, global warming and pollution in natural water resources indicate that there will be a water crisis in the world of the future. Water resources vital to life must be protected for health, nutrition and livelihood. This study points to heavy metal pollution in natural water resources and the toxic effects of this pollution on a bio-indicator organism in the food chain. In order to prevent this toxicity, which can reach animals and humans through the food chain, water resources should be given importance and protected by legislation. In addition, solution strategies that will reduce water pollution and prevent toxic effects should be kept on the agenda.

## Data Availability

The datasets used and/or analyzed during the current study are available from the corresponding author on reasonable request.
